# 

**Published:** 1877-11

**Authors:** 


### BOOKS AND PAMPHLETS RECEIVED.

Cyclopædia of the Practice of Medicine. Edited by Dr. H. Von Ziemssen, Professor of Clinical Medicine, Munich, Bavaria. Vol. XVI. Diseases of the Locomotive Apparatus, and General Anomalies of Nutrition. By Prof. H. Senator, of Berlin; Prof. E. Seitz, of Giessen; Prof. H. Immermann, of Basel; and Dr. Birch-Hirschfeld, of Dresden. William Wood & Co. 1877. Octavo, pp. 1,060.

The Ear: Its Anatomy, Physiology, and Diseases. A Practical Treatise for the Use of Medical Students and Practitioners. By Charles H. Burnett, A. M., M D., etc., etc. With 87 illustrations. Philadelphia: Henry C. Lea. Octavo; cloth. 1877; p. 606.

Forensic Medicine and Toxicology. By W. Barthust Woodman, M. D., F. R. S., and Charles Meymott Tidy, M. B. F. R. S. With eight full-page lithographic plates, and one hundred and fifteen other illustrations. Philadelphia: Lindsay & Blakiston. 8vo. 1877; pp. 1,083.

Practical Hints on the Selection and Use of the Microscope. Intended for beginners. By John Phin, editor *American Journal of Microscopy*. Second edition. New York Industrial Publication Company. 12mo.

Lectures on Practical Surgery. By H. H. Toland, M. D., Professor of the Principles and Practice of Surgery and Clinical Surgery in the Medical Department of the University of California. With numerous illustrations. Philadelphia: Lindsay & Blakiston. 8vo. 1877. pp. 506.

Transactions of the Medical Association of Georgia. Twenty-eighth Session. 1877.

Transactions of the Minnesota State Medical Society. 1877.

Annual Report of the City Registrar, of the Births, Deaths, and Marriages in the city of Boston, for the year 1876.

A New Method of Treating Fracture of the Clavicle. By Henry Van Buren, M. D.

Address delivered at the Third Annual Meeting of the Association of Alumni, and Officers of the Medical Department of the University of Buffalo. By Frank H. Hamilton, A. M., M. D.

Delaware State Medical Society. Session at Dover, Delaware, June, '77. Address of the President, John J. Black, M. D.

Long Island College Hospital. Annual Announcement and Circular. 1877–78.

École de Médecine et de Chirurgie de Montreal. Faculté Médicale de l' Université Victoria. Session 1877–78.

Changes in New England Population. By Nathan Allen, M. D.

Hospitals: their History, Organization, and Construction. Boylston Prize Essay of Howard University for 1876. By W. Gill Wylie, M. D. New York: D. Appleton & Co. 1877. 8vo. Cloth. pp. 326.

### ANNOUNCEMENTS FOR THE MONTH.

### SOCIETY MEETINGS.

Chicago Medical Society—Mondays, Nov. 5 and 19.

Chicago Society of Physicians and Surgeons—Mondays, Nov. 12 and 26.

### CLINICS.

MONDAY.

Eye and Ear Infirmary—2 to 4 P. M., by Prof. Holmes and Dr. Hotz.

Mercy Hospital—2 to 3 P. M. Surgical, by Prof. Andrews.

Rush Medical College—1:30 P. M. Medical, by Dr. Bridge.

County Hospital—8 P. M. Necropsy, by Dr. Danforth.

Woman's Medical College—3 P. M. Surgical, by Prof. Owens.

TUESDAY.

County Hospital—1:30 P. M. Medical, by Prof. Bevan; 2:30 P. M. Surgical, by Dr. Bogue.

Mercy Hospital—2 P. M. Medical, by Prof. Hollister.

Eye and Ear Infirmary—2 P. M. Prof. Jones.

WEDNESDAY.

County Hospital—1:30 P. M. Gynecological, by Prof. Fitch; 2:30 P. M. Ophthalmological, by Dr. Montgomery.

Mercy Hospital—2 P. M. Eye and Ear, by Prof. Jones.

Rush Medical College—4 P. M. Diseases of the Chest, by Prof. Ross.

THURSDAY.

Mercy Hospital—2 P. M. Medical, by Prof. Davis.

Rush Medical College—1:30 P. M. Neurological, by Prof. Lyman.

Eye and Ear Infirmary—2 to 4 P. M. Operations, by Prof. Holmes and Dr. Hotz.

FRIDAY.

Mercy Hospital—2 P. M. Medical, by Prof. Davis.

County Hospital—1:30 P. M. Medical, by Prof. Ross; 2:30 P. M., Surgical, by Prof. Gunn.

Woman's Medical College—10 P. M. Ophthalmological, by Dr. Montgomery.

SATURDAY.

Rush Medical College—2 P. M. Surgical, by Prof. Gunn.

Chicago Medical College—2 P. M. Surgical, by Prof. Andrews and Isham; 3 P. M., Diseases of Chest, by Prof. Johnson.

Woman's Medical College—12 M. Gynecological, by Prof. Fitch; 3 P. M. Dermatological, Dr. Maynard.

Special Clinics daily, from 2 to 4 P. M., at the South Side Dispensary, and at the Central Free Dispensary.

For schedule of lectures at the colleges, apply to the college janitors.

